# Protective or harmful? A qualitative exploration of older people’s perceptions of worries about falling

**DOI:** 10.1093/ageing/afac067

**Published:** 2022-04-01

**Authors:** Toby J Ellmers, Mark R Wilson, Meriel Norris, William R Young

**Affiliations:** School of Sport and Health Sciences, University of Exeter, UK; The College of Health, Medicine and Life Sciences, Brunel University London, UK; Neuro-otology Unit, Department of Brain Sciences, Imperial College London, UK; School of Sport and Health Sciences, University of Exeter, UK; The College of Health, Medicine and Life Sciences, Brunel University London, UK; School of Sport and Health Sciences, University of Exeter, UK; The College of Health, Medicine and Life Sciences, Brunel University London, UK

**Keywords:** fear of falling, anxiety, balance confidence, falls, qualitative, older people

## Abstract

**Background:**

worries about falling are common in older people. It has been suggested that these worries can reduce balance safety by acting as a distracting dual-task. However, it is also possible that worries may serve a protective purpose. The present work adopted a qualitative approach to conduct an in-depth exploration of older people’s experiences of worries about falling.

**Methods:**

semi-structured interviews were conducted with 17 community-dwelling older people (mean age = 79 years; males = 5/17) who reported experiencing worries about falling. Reflexive thematic analysis was used to analyse the data.

**Results:**

experiencing a fall—or otherwise recognising one’s balance limitations—brought the physical realities of participants’ ageing bodies to the forefront of their awareness. This led to the recognition of their susceptibility for an injurious fall, which triggered worries about falling in situations that threatened their balance. When preventing the subject of their worries (i.e. an injurious fall) was perceived to be within the individual’s locus of control, worries led to protective adaptations to behaviour. In contrast, when the subject of their worries was perceived to be outside their control, worries triggered feelings of panic—leading to unhelpful changes in behaviour.

**Conclusion:**

these findings provide novel insight into the development and consequences of worries about falling in older people. They highlight the importance of considering an individual’s perception of control before deciding to clinically intervene to reduce worries about falling.

## Key Points

- We adopted a qualitative approach to explore older people’s experiences of worries about falling.
- Our findings highlight perceived control as a crucial factor in determining whether worries are protective or maladaptive.
- High perceptions of control meant that worries led to protective behavioural adaptations.
- In contrast, low perceived control meant that worries triggered a negative spiral of panic and unhelpful changes in behaviour.
- Clinicians should assess the presence of worry and whether the subject of worry (i.e. injurious fall) is perceived as controllable.

## Introduction

Researchers have long assumed a relationship between fear of falling and increased risk of falls [1]. Traditional conceptualisations of this relationship are based on the notion that fearful individuals are more likely to avoid activities in which they could fall (e.g. [2]). As such, these individuals are proposed to become deconditioned to performing activities of daily living, resulting in muscle atrophy, poorer balance and increased risk for falls. Recent research has, however, questioned the extent to which fear of falling predicts future falls [3–5], whilst other findings suggest that this relationship may only be present in certain individuals (e.g. those with balance/gait impairments [6]). This conflicting evidence likely reflects the potential for fear of falling to affect fall-risk in both adaptive (e.g. avoiding an activity in which a fall is likely to occur) and maladaptive ways (e.g. excessively avoiding ‘all’ activity) [7].

Whilst fear of falling may have both adaptive and harmful consequences, anxiety is emerging as a seemingly maladaptive construct with respect to balance and fall-risk [8–10]. Although related, fear and anxiety are distinct [11–13]. Fear reflects the emotional response when an individual perceives that imminent harm (i.e. a fall) ‘is likely’ to occur. In contrast, anxiety is a future-orientated emotional state, characterised by ‘what if’ thoughts about a potentially harmful event that may-or-may-not occur (e.g. persistent worries about the injuries that one may sustain ‘if’ they were to fall) [11–13]. Such worries are argued to both be a core feature of anxiety and also serve to propagate further anxious feelings [12].

Older people frequently worry about falling. We have previously reported that around 50% of older people will experience worrisome thoughts when they feel that their balance is threatened and that a fall is likely to occur [14]. The consequences of such worries on behaviour are, however, unknown. It has been suggested that attending to worrisome thoughts whilst walking may reduce safety by acting as a distracting ‘dual-task’ [14]. This supports psychological research that describes how worrisome thoughts can distract attention and impair performance on a range of tasks [12, 15]. It is, however, also possible that worries about falling may serve a protective purpose [16]. For example, they may enhance the detection of potential threats to balance or ensure that appropriate behavioural adaptations are made to reduce the likelihood of a loss of balance occurring (e.g. increasing step clearance over an obstacle [17, 18]).

Our previous work sheds some light on the (threatening) situations that can trigger worries about falling in older people [14]. However, less is known about the ways, and reasons why, such worries develop in the first place—in addition to whether older people view these thought processes as having an overall protective or detrimental effect on their balance safety. Developing a detailed and nuanced understanding of what worries about falling mean to the older people that experience them is crucial before recommendations can be made regarding if—and how—such worries should be clinically managed. Whilst previous qualitative research has provided detailed insight into older people’s perceptions of fear of falling [19, 20], a thorough exploration of worrisome thoughts about falling has yet to be conducted. Therefore, the present work adopted a qualitative approach to conduct an in-depth exploration of older people’s perceptions and experiences of worries about falling. Specifically, we sought to understand why such worries arise in the first place, as well as what function these worries serve and how they are perceived to affect balance and safety.

## Methods

Ethical approval for the study was obtained from the local ethics committee and the research was carried out in accordance with the Declaration of Helsinki.

### Participants

Seventeen community-dwelling older people who self-identified as experiencing worries about falling participated in the research. The study was advertised through a UK-wide network of locally run interest groups for older people, and interested individuals were invited to participate if they were aged 60 years or older and answered ‘Yes’ to the following question: ‘Do you experience worries about falling?’ Interested participants contacted the first author, who then provided them with written information about the study. Participants were encouraged to ask any questions before agreeing to take part, and all participants provided written informed consent prior to participation.

### Interviews

The first author (a postdoctoral research fellow with a background in psychological factors influencing balance and falls) conducted semi-structured interviews with individual participants. Due to restrictions on face-to-face data collection following the outbreak of covid-19, interviews were instead conducted either over telephone (*n* = 9) or video-call platform (*n* = 8), based on participants’ personal preference. All interviews were conducted whilst participants were sitting alone in a quiet room in their homes. To both capture the unique experiences of individual participants and ensure that the key research topics were covered, the first author developed a semi-structured interview guide, with consultation from co-authors (see Supplementary data). Questions focussed on how and why worries about falling develop, when they occur, as well as the consequences that individuals believe these worries have for them, with respect to mood, broader psychological functioning, behaviour and any compensatory strategies used to overcome worries (if relevant). Whilst the interview guide was used as a reminder of the broader topics to discuss, the specific questions asked were determined based on what each individual participant discussed. Interviews lasted an average of 42 min (range: 31–64 min). Interviews were audio-recorded and transcribed verbatim. Prior to the interview, participants reported demographic information and also completed the Falls Efficacy Scale-International short-form (FES-I [21]) as a measure of generalised concerns about falling.^1^

### Analysis

Data were analysed thematically, using the reflexive approach developed by Braun and Clarke [22, 23]. We selected this approach as we sought to identify themes and patterns of meaning across the dataset. Reflexive thematic analysis begins with data familiarisation, followed by extensive coding of the data; searching for themes; reviewing and revising themes and defining and naming themes. An inductive approach was adopted throughout the analysis process. Complete coding was utilised, and data were coded based on both semantic (i.e. descriptive) and latent (i.e. interpretative) meanings. Related codes were grouped together under central organising concepts, which then became candidate themes. Candidate themes were reviewed against coded data extracts and the dataset as a whole and revised accordingly (see Supplementary data). Whilst the analysis process was led by the first author, theme development, review and revision took place with consultation from co-authors. To further maximise rigour and reliability, the first author also kept a self-reflective journal/diary throughout the research that listed, among other things, his assumptions regarding worries about falling and any salient ‘noticings’ throughout the data collection or analytical process.

## Results

Participants had a mean age of 79 years (range: 75–90 years). Five out of the 17 participants were male. There were no diagnoses of dementia or any other degenerative neurological disease reported in any of the participants. Every participant lived in the community, and the majority of whom (10/16; data missing for one participant) lived alone. Nine participants (out of 16; data missing for one participant) reported that they had fallen at least once in the previous year, and six participants (out of 16; data missing for one participant) reported that they have difficulty walking one-quarter of a mile. All participants reported that they could perform basic activities of daily living without assistance, and every participant reported their overall health as good, very good or excellent. FES-I [21] scores ranged from 8 to 17, with a mean score of 12.5. This indicates an overall high level of concern about falling in our sample (when using the validated scores of 7–10 and 11–28 to indicate low and high concerns, respectively [24]). Full demographic and background information for the sample is reported in Table 1.

**Table 1:** Participant background and demographic data

**Pseudonym** ^**a**^	**Age**	**Gender**	**Living arrangement**	**Fallen in past 12 months**	**Difficulty walking ¼ mile**	**Walking aid**	**Assistance for Activities of Daily Living (ADL)**	**Self-rated health**	**Short FES-I scores**
Thomas	77	Male	With a partner/family member	More than once	No	No	No	Good	11
Robert	75	Male	Alone	Once	No	No	No	Very good	12
Patricia	76	Female	^*^	^*^	^*^	^*^	^*^	^*^	15
Carol	90	Female	Alone	No	Yes	Walking stick/cane	No	Very good	16
Judith	73	Female	With a partner/family member	No	Yes	No	No	Good	11
Barbara	78	Female	Alone	More than once	Yes	No	No	Good	17
Edward	83	Male	Alone	No	No	Walking stick/cane	No	Good	12
Linda	78	Female	With a partner/family member	No	No	No	No	Very good	10
Joyce	77	Female	Alone	No	No	No	No	Excellent	9
Dorothy	77	Female	With a partner/family member	More than once	Yes	Walker	No	Very good	17
Helen	84	Female	Alone	No	Yes	Walker	No	Good	17
Gloria	81	Female	Alone	More than once	Yes	No	No	Excellent	14
Margaret	73	Female	With a partner/family member	More than once	No	No	No	Good	9
Charles	75	Male	Alone	More than once	No	No	No	Good	10
Frank	80	Male	Alone	No	No	No	No	Very good	13
Betty	86	Female	Alone	Once	No	Walking stick/cane	No	Very good	8
Shirley	75	Female	With a partner/family member	More than once	No	No	No	Excellent	11

^a^Pseudonyms were randomly selected from a list of most popular names from the decade in which the participant was born.

^*^Participant missing data for this variable.

Short FES-I is scored from 7–28, with higher scores indicating greater concerns about falling. Scores between 7–10 and 11–28 indicate low and high levels of concerns, respectively [19].

The data analysis process resulted in the generation of four themes: (i) ‘The age of falling’: recognition of the ageing body; (ii) in control of being careful: worries as a protector (two subthemes: ‘Identifying the risks and planning for safety’ and ‘Consciously engaging movement strategies’); (iii) uncertain and out of control: worries as a source of panic and (iv) ‘A prisoner in the house’: activity curtailment and an altered sense of self. The following section will present each of these themes, in turn, with the support of pseudonymised quotes.

### ‘The age of falling’: recognition of the ageing body

It was clear across the dataset that worries about falling were a direct response to individuals perceiving themselves as being susceptible to falls. In almost all cases, this perceived vulnerability was triggered by a fall itself and was seen as an ‘inevitable’ part of the ageing process. As Patricia stated: ‘With regards to these two incidents [falls], I just thought, oh dear, you’ve reached the age of falling’.

Participants described their previous fall/s in vivid detail; with this memory (or memories) replaying when they became worried about falling. In the few participants who had not experienced a fall, ‘near misses’ and/or bouts of unsteadiness nonetheless resulted in increased awareness of their worsening balance. Participants appeared to have viewed their balance as unproblematic—almost taking it for granted—until these falls (or near misses/bouts of unsteadiness) occurred. These experiences then brought the physical realities of their ageing body to the forefront of their awareness.

Individuals also recognised that the ageing process had made them more vulnerable to harm occurring if they were to fall. As Charles described:

I’ve seen people ageing all my life and I’ve heard old people talking about, ‘Oh, that was when I had my first fall’, as if it was inevitable when you get old. But I’ve never identified myself as being somebody that’s going to be suffering from the consequences of old age the way other human beings have. But when it starts to happen to you, to oneself, one has to acknowledge that one is fitting into a pattern here that you’ve been aware of in other people for many years and now you are fitting into it. And if you’re going to be the sort of person that starts having falls, the next thing that happens to old people is that they break bones when they have falls.

As illustrated above, vicarious experience appeared to play a key role in participants recognising the increased risk of harm posed by falling. Other participants reported observing how friends and family members had experienced serious injury and even death following a fall. This seemed to directly inform the specific worries that participants had, with most worries relating to the injuries that they believed they would sustain if they were to fall. Most worries, however, related not to the physical pain of an injury itself, but to how an injury would impact their ability to continue living a rich and fulfilling life. As Linda articulated: ‘My time is shorter than it used to be. I don’t want to waste my time lying on the settee with a broken leg’.

Some participants worried about being unable to maintain a physically active and enriching lifestyle, whilst others worried about becoming socially isolated following an injury. Most participants lived alone, and worries about losing one’s independence following an injurious fall were common threads running throughout their narratives. For example, Patricia highlighted:

[I worry about] hurting myself, causing an ongoing problem if I hurt myself […] That would have a knock-on problem, that would cause problems, not just for me but for those who will have to accommodate that to a certain extent, you know? That is a bit of a worry. Not being able to cope […] Not being able to do what I normally do which is be independent actually, you know? Live totally independently.

As illustrated above, participants also worried about how a fall would impact the lives of those that would have to care for them if they were to injure themselves. Participants viewed falls as having potentially life-changing consequences for both themselves and those close to them. It is, therefore, perhaps unsurprising that increased awareness of one’s susceptibility to falls resulted in worries about these negative outcomes occurring.

### In control of being careful: worries as a protector

Worries about falling were most frequently reported in situations where participants perceived their balance to be threatened. Often, these were similar situations in which they had previously fallen (or lost their balance and nearly fallen). Participants also described how they would frequently experience worries when preparing to engage in a situation which they believed would threaten their balance; for instance, when walking along a flat path that they knew led to a steep hill.

Nonetheless, most participants described the worries about falling that they experienced as having a protective effect, with worrisome thoughts drawing attention towards potential risk. Participants discussed the falls that they had experienced—and the subsequent worries that they triggered—as ‘wake-up calls’, which led to enhanced attention directed towards ‘being careful’. Specifically, worries led to a two-pronged approach designed to minimise the risk of falling: (i) identifying the risks and planning for safety and (ii) consciously engaging movement strategies.

### Identifying the risks and planning for safety

In anticipation of performing a potentially challenging or dangerous task, individuals described how worrisome thoughts encouraged them to identify and assess potential risks. As Charles stated: ‘That’s what you’re concentrating on; assessing the risk and planning how you’re going to counter that risk and weighing up the consequences’. When the upcoming risk was perceived as one that could be minimised through behavioural modification (i.e. the risk was perceived to be one which they could control), then pre-emptive preparatory strategies were adopted to enhance safety. Dorothy likened this process to ‘an army campaign in my head’. Common preparatory strategies included wearing ‘sensible’ shoes and scanning one’s environment to identify potential tripping hazards prior to setting off. Sometimes the situation was evaluated as being ‘too risky’ and a fall deemed ‘too likely’. In these instances, pre-emptive preparatory strategies instead involved identifying alternative routes to avoid the potential threat (e.g. a steep hill) without having to avoid the activity (e.g. going on a country walk) altogether. As Edward highlighted:

I would make a judgement as to whether it was too difficult for me and if I couldn’t really see myself going down there I would have to find a different route or something like that but I wouldn’t risk falling.

Whilst such risk assessment largely occurred prior to performing the potentially threatening task, some participants described how they would continue to update their judgements during the task itself. For example, Linda reported that she would evaluate her balance/level of stability throughout the ongoing task to determine her level of safety and identify whether she needed to either find an alternative route (as described above) or consciously alter her movement strategy (see below subtheme).

Once participants had identified and evaluated the risk, this then allowed them to make preparations to negotiate the threatening scenario. These preparations also appeared to reduce the likelihood that individuals would find themselves worrying when performing the task itself, as Shirley described: ‘It lessens the worry if you feel more prepared by checking out where you’re walking’.

### Consciously engaging movement strategies

Almost every participant described consciously engaging in strategies during the task itself to minimise the likelihood of falling. Participants highlighted two main strategies. The first involved looking down at the ground whilst walking to reduce the risk of tripping, as Judith described: ‘I’m always looking down because I’m looking for uneven ground or anything because I’m frightened of tripping’. The second strategy involved slowing down and consciously controlling the walking pattern, as described by Shirley: ‘I’m really quite conscious of not feeling very steady. So, as far as the walking goes, I just try to think about putting my heel down first, you know, because I’m afraid of catching my toe’. These quotes illustrate that participants were aware of what they needed to do to prevent the fall occurring (e.g. look down at the ground or pick up their feet) and thus consciously engaged the appropriate movement strategy required to maintain safety.

These conscious strategies were seemingly employed as participants viewed preventing an injurious fall as being within their control—further emphasising the protective element of worries. As Charles identified: ‘I’m still at the stage where I believe that if I concentrate and apply myself to the task, I will be able to do it without the fall’. Like most others, Charles believed that preventing a fall was within his locus of control. For most participants worrisome thoughts thus had a protective effect, increasing concentration on the task at hand and ensuring that the correct motor pattern necessary for maintaining safety was (consciously) engaged.

### Uncertain and out of control: worries as a source of panic

In contrast, a minority of participants identified worries about falling as being ‘unhelpful’ with respect to their safety. These negative feelings seemed to stem from individuals being either uncertain of the causes of their fall/instability or deeming the causes to be outside of their control. This resulted in the perception of a lack of control over preventing the subject of their worry—an injurious fall—from occurring. For example, Betty had experienced multiple falls that were a consequence of other pedestrians knocking into her. This led her to worry and subsequently ‘freeze’ whenever she was walking in a crowded area. She also described finding herself becoming distracted by approaching pedestrians, which meant that she was less able to focus on strategies that she believed were crucial for ensuring her safety (e.g. looking down at the ground to avoid tripping; see above theme). Similarly, Frank reported frequent—yet unpredictable—bouts of unsteadiness that were outside of his immediate control and left him worried about falling.

Across these participants, this perceived lack of control caused significant distress, leading to intense feelings of panic in situations that triggered worries about falling. As Judith described:

I went for a walk a few weeks ago and there’s a particularly steep hill, it wasn’t slippy or anything, but it’s very steep and I was holding onto the wall at the side coming down. I don’t feel safe just free when I’m walking sort of down like that. I feel propelled forward somehow. I have that feeling, a bit panicky, and I think that’s because of that experience of coming off that treadmill. I can still feel how that felt then, that I hadn’t got control. […] It’s just like a really frightening feeling that everything’s going to end. It’s scary, it’s a real panic.

Feeling ‘out of control’ and ‘expecting the worst’ were common threads running through these participants’ narratives. As illustrated above, a lack of perceived control over one’s body during scenarios that threaten balance seemed to trigger an impending sense of inevitable catastrophe. Thus, rather than worrisome thoughts triggering behavioural adaptations that minimise the risk of falling (as described in the previous theme), these participants would instead panic and alter their behaviour as if preparing to fall. As Dorothy, who experienced worries due to frequent falls caused by a ‘randomly’ dislocating knee, described:

I start panicking. You know, I see this hill going down and I just say to my husband, ‘I’m not going. I just can’t go down there’. I won’t go down there and I just don’t see why I should struggle to go down there on, you know, step-by-step-by-step, inch-by-inch. Because that’s how freaked out I get. I’m just anticipating I’m going to fall every time I make a step. […] I just completely go into a sort of, you know, bent over stance. I can’t—I couldn’t possibly stand up straight. I’m stiff and tense and leaning over. My husband says, you know, ‘Stand up, stand up. You will fall if you carry on like that’, you know, but I just go into a complete panic about falling again.

Whilst Judith reported how the feelings of panic that she experienced led her to hold onto the wall for protection, Dorothy appeared to select a potentially inappropriate protection strategy that paradoxically seemed to increase the risk of the ‘anticipated’ fall occurring. Similarly, participants also explained how feelings of panic led them to often ‘hesitate’ in situations where they perceived their balance to be threatened. Such hesitancy/cautiousness was identified by participants as increasing the likelihood of an incorrect ‘reaction’, thus reducing safety.

### ‘A prisoner in the house’: activity curtailment and an altered sense of self

Whilst most participants reported adapting their behaviour in situations where they worry about falling (as described in Theme 2), a smaller number of participants avoided these situations altogether. Such activity curtailment was usually described in ways implying that it was excessively cautious. For example, numerous participants identified that their worries made them reluctant to try and ‘push’ themselves and thus avoided activities that they ‘probably’ could engage in without experiencing a fall.

Participants highlighted that avoiding activities because they were worried about falling altered the way that they viewed themselves, making them feel ‘inadequate’, ‘older’ and ‘useless’. Participants spoke about how their worries ‘closed the doors’ on certain activities that they wished to engage in. There was a reduced number of ‘safe’ activities open for them to engage in, and this led to the realisation that their life was ‘shrinking’. For example, Carol described how her worries had stopped her from travelling to see her friend in another city:

It’s stopping me doing something I really want to do because I’m afraid of what might happen. […] It makes me feel weak and cowardly and I don’t want to tell my friends. But it also makes me realise how much my life has shrunk.

Activity curtailment appeared to be closely linked to participants’ sense of themselves, and their reasons for being. Participants who reported that they continued to engage in activities despite their worries appeared to do so in order to maintain their sense of being. As Robert described:

I still want to walk out and about because I don’t want to be a prisoner in the house. I suppose if you want to eliminate the concern you just don’t do it but I wouldn’t want to do that; then life’s not worth living for me.

An individual’s level of resilience appeared to be a key factor in whether the worries experienced led to activity curtailment or not. A common thread underpinning this resilience appeared to be the upbringing that participants experienced in which they learned to ‘just get on with it’. Participants spoke about how their upbringing provided ‘reserves’ and ‘resources’ that they could draw on when worried. Interestingly, whilst Carol reported how her worries had recently prevented her from travelling to visit her friend (as described above), she nonetheless drew on these ‘reserves’ to minimise the overall impact of her worries:

When I was a child during the war, I walked alone in the blackout, you’re too young to remember that, but you walked outside fearlessly […] So I think there’s maybe a shred of that kind of, the way I tell myself just not to panic and go on and do what I’ve got to do.

As illustrated above, Carol drew on historical experiences to help maintain her sense of self in the face of the worries she experienced and the resulting activity avoidance. A number of other participants similarly reported drawing on salient ‘overcoming’ narratives from their past (particularly those from their childhood) when they found themselves becoming worried about falling. This appeared to provide them with the confidence needed to believe that they could also control—and overcome—the worries about falling that they experienced, thus avoiding the need to curtail activities in response to such feelings.

## Discussion

This present work adopted a qualitative approach to enhance our understanding of older people’s experiences of worries about falling. Specifically, we sought to understand why such worries arise in the first place, as well as what function these worries serve and how they affect balance and safety.

Our findings highlight that worries about falling develop in response to an individual recognising their vulnerability for an injurious fall. Participants had previously viewed their balance as unproblematic—almost taking it for granted—until a fall (or ‘near misses’ and/or bouts of unsteadiness) occurred. This supports previous qualitative research that reported how older people will often view themselves as ‘the type that doesn’t fall’, until they do [25]. In the present research, increased awareness of one’s risk of falling brought the physical realities of their ageing body to the forefront of their awareness. Worries about falling were then triggered in situations where they perceived their balance to be threatened. Vicarious experience of having observed friends and family members becoming seriously injured from a fall fed into the specific worries experienced; with most participants worrying about how an injury would impact their ability to live an independent, fulfilling life. This complements previous qualitative research that has reported similar findings with respect to the feared consequences of falling [19, 26].

Our findings also highlight the need to nuance the prevailing perception that anxiety and associated changes in attention, i.e. worrisome thoughts, are inherently maladaptive (e.g. [9]). In general, worries (a key component of anxiety) motivated individuals to modify or adapt the activities they engaged in (e.g. find an alternative route that bypassed steep hills/uneven terrain)—rather than avoiding these potentially dangerous activities altogether. Previous qualitative research has similarly reported that older adults who perceive themselves to be at an increased risk of falling tend to frequently modify their behaviour during potentially risky activities rather than completely avoiding them [26]. Based on the data presented, we propose that the presence of worry itself is unlikely to be a cause for concern—given that most participants described how these worries serve some degree of protective purpose. Participants reported how these worries enhanced their concentration on the task at hand and resulted in (consciously initiated and controlled) behavioural adaptations that they believed were integral for ensuring safety. This supports previous laboratory-based research that describes how older adults will report directing conscious attention towards controlling their walking pattern when their balance is threatened [27]. Whilst researchers have proposed that such conscious control may disrupt walking behaviours and increase falls [28], our present findings identify an adaptive role of this motor control strategy.

We have previously suggested that worries about falling may reduce balance safety in older people [14]. Whilst most participants viewed worries as serving a protective function, our findings highlight that worries that lead to panic (defined as a strong, uncontrollable emotional reaction that overwhelms logical thought and behaviour [29]) reflect a maladaptive cognitive process—triggering intense feelings of distress and ‘unhelpful’ behavioural adaptations likely to reduce safety. We suggest that such feelings of panic likely underpin the maladaptive changes in behaviour previously observed in ‘certain’ older people when their balance is threatened [10, 27, 30], e.g. overly cautious gait [30] or failures to sufficiently plan future stepping actions [10, 27].

**Figure 1:**
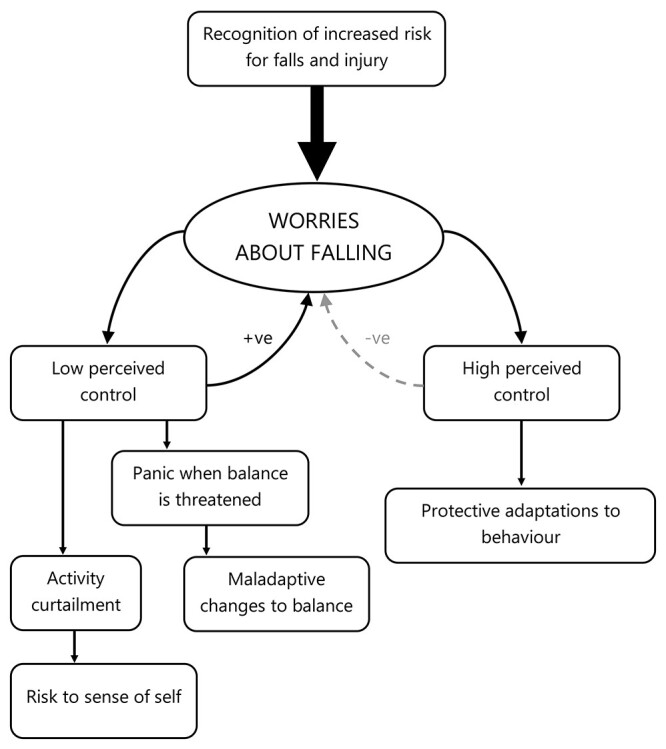
Conceptual framework describing the origination and consequences of worries about falling.

What determines whether worrisome thoughts about falling serve a protective or maladaptive function? As presented in the conceptual framework derived from our data (see Figure 1), we propose that an individual’s perception of control is the crucial determining factor (or the ‘fork in the road’). If individuals perceive the subject of their worries (i.e. the injurious fall) as being within their locus of control to prevent, then worries themselves will encourage positive, protective changes to behaviour (e.g. [26]). In contrast, if preventing the subject of their worries is perceived to be outside of their control, then feelings of panic will be triggered. This will then lead to a spiral of persistent and pervasive worry that will disrupt the behavioural adaptations necessary for ensuring safety. It is important to note that these perceptions of control are context-dependent rather than generalised. For example, when walking across a familiar but uneven path, an individual may believe that they have control over preventing the subject of their worries (i.e. an injurious fall) from occurring. However, if these same worries were to then occur in, for example, a context in which this individual had previously observed someone having an injurious fall, then their perceptions of control over preventing this happening to themselves may be low.

Our proposal that it is not the presence of worries per se that determines their effects on behaviour, but rather one’s perception of control with regards to these thoughts, aligns with work from psychology [31]. For instance, Weiner’s Attribution Theory [32] posits that attributing outcomes to controllable causes leads to greater motivation for achieving future success (i.e. greater motivation to change behaviour to avoid a future fall). The present findings add to the existing body of research that highlights the positive effect that high perceptions of control can exert over older people’s mental and physical wellbeing [33].

Based on these findings, we recommend that clinicians working with older people with balance problems should assess the extent to which these individuals worry about falling. Such assessment can be easily achieved through using the (three-item) ‘fall-related ruminations’ sub-scale of the Gait-Specific Attentional Profile [34]. A short discussion will then allow the clinician to determine (i) what situations/contexts the individual finds themselves worrying in and (ii) the extent to which the subject of these worries is perceived to be controllable or not. This will help to identify the individuals most at risk of adopting maladaptive behaviours when their balance is threatened. The underlying driver of any perceptions of low control should then be probed and targeted through a therapeutic intervention aimed at improving the individual’s sense of control in a given task/scenario. For example, an individual that has low perceived control due to having experienced falls caused by pedestrians knocking into them could be prescribed training to enhance balance recovery following unpredictable external perturbations.

## Conclusion

These findings provide novel insight into the development and consequences of worries about falling in older adults. When preventing the subject of their worries (i.e. an injurious fall) was perceived to be within the individual’s locus of control, worries led to protective adaptations to behaviour. In contrast, when preventing the subject of their worries was perceived to be outside of their control, then a negative cycle of panic and persistent, ruminative worries was triggered—leading to changes in balance likely to reduce safety. These findings highlight the importance of considering an individual’s perception of control before deciding to clinically intervene to reduce worries about falling.

## Supplementary Material

aa-21-1751-File003_afac067

## Footnotes

1 We acknowledge that asking participants to complete the FES-I prior to participation could have primed them to think about worries within the context of the scenarios described in this scale. However, as participants tended to describe worries directly related to falls that they had personally experienced, we do not deem this likely to have introduced a major confound.

## References

[1] Friedman SM, Munoz B, West SK, Ruben GS, Fried LP. Falls and fear of falling: which comes first? A longitudinal secondary prevention. J Am Geriatr Soc 2002; 50: 1329–35. 12164987 10.1046/j.1532-5415.2002.50352.x

[2] Brummel-Smith K . Falls in the aged. Prim Care 1989; 16: 377–93. 2664838

[3] Clemson L, Kendig H, Mackenzie L, Browning C. Predictors of injurious falls and fear of falling differ: an 11-year longitudinal study of incident events in older people. J Aging Health 2015; 27: 239–56. 25117181 10.1177/0898264314546716

[4] Pohl P, Ahlgren C, Nordin E, Lundquist A, Lundin-Olsson L. Gender perspective on fear of falling using the classification of functioning as the model. Disabil Rehabil 2015; 37: 214–22. 24786969 10.3109/09638288.2014.914584 PMC4364267

[5] Weijer RHA, Hoozemans MJM, Meijer OG, van Dieën JH, Pijnappels M. The short- and long-term temporal relation between falls and concern about falling in older adults without a recent history of falling. PLoS One 2021; 16: e0253374. 34242215 10.1371/journal.pone.0253374 PMC8270453

[6] Allali G, Ayers EI, Holtzer R, Verghese J. The role of postural instability/gait difficulty and fear of falling in predicting falls in non-demented older adults. Arch Gerontol Geriatr 2017; 69: 15–20. 27866086 10.1016/j.archger.2016.09.008 PMC5186402

[7] Delbaere K, Close JCT, Brodaty H, Sachdev P, Lord SR. Determinants of disparities between perceived and physiological risk of falling among elderly people: cohort study. BMJ 2010; 341: c4165. 20724399 10.1136/bmj.c4165 PMC2930273

[8] Hallford DJ, Nicholson G, Sanders K, McCabe MP. The association between anxiety and falls: a meta-analysis. J Gerontol Ser B 2017; 72: 729–41. 10.1093/geronb/gbv160 26791279

[9] Young WR, Williams MA. How fear of falling can increase fall-risk in older adults: applying psychological theory to practical observations. Gait Posture 2015; 41: 7–12. 25278464 10.1016/j.gaitpost.2014.09.006

[10] Young WR, Wing AM, Hollands MA. Influences of state anxiety on gaze behavior and stepping accuracy in older adults during adaptive locomotion. J Gerontol B Psychol Sci Soc Sci 2012; 67 B: 43–51. 21808071 10.1093/geronb/gbr074

[11] Borkovec TD, Robinson E, Pruzinsky T, DePree JA. Preliminary exploration of worry: some characteristics and processes. Behav Res Ther 1983; 21: 9–16. 6830571 10.1016/0005-7967(83)90121-3

[12] Eysenck MW, Derakshan N, Santos R, Calvo MG. Anxiety and cognitive performance: attentional control theory. Emotion 2007; 7: 336–53. 17516812 10.1037/1528-3542.7.2.336

[13] LeDoux JE, Pine DS. Using neuroscience to help understand fear and anxiety: a two-system framework. Am J Psychiatry 2016; 173: 1083–93. 27609244 10.1176/appi.ajp.2016.16030353

[14] Ellmers TJ, Cocks AJ, Young WR. Exploring attentional focus of older adult fallers during heightened postural threat. Psychol Res 2020; 84: 1877–89. 31119367 10.1007/s00426-019-01190-6 PMC7479009

[15] Wilson M . From processing efficiency to attentional control: a mechanistic account of the anxiety performance relationship. Int Rev Sport Exerc Psychol 2008; 1: 184–201.

[16] Litwin H, Erlich B, Dunsky A. The complex association between fear of falling and mobility limitation in relation to late-life falls: a SHARE-based analysis. J Aging Health 2018; 30: 987–1008. 28553817 10.1177/0898264317704096 PMC6655432

[17] Brown LA, Doan JB, McKenzie NC, Cooper SA. Anxiety-mediated gait adaptations reduce errors of obstacle negotiation among younger and older adults: implications for fall risk. Gait Posture 2006; 24: 418–23. 16420978 10.1016/j.gaitpost.2005.09.013

[18] McKenzie NC, Brown LA. Obstacle negotiation kinematics: age-dependent effects of postural threat. Gait Posture 2004; 19: 226–34. 15125911 10.1016/S0966-6362(03)00060-2

[19] Tischler L, Hobson S. Fear of falling: a qualitative study among community-dwelling older adults. Geriatr Nurs 2018; 23: 37–53.

[20] Lee F, MacKenzie L, James C. Perceptions of older people living in the community about their fear of falling. Disabil Rehabil 2008; 30: 1803–11. 19031207 10.1080/09638280701669508

[21] Kempen GIJM, Yardley L, Van Haastregt JCM et al. The short FES-I: a shortened version of the falls efficacy scale-international to assess fear of falling. Age Ageing 2008; 37: 45–50. 18032400 10.1093/ageing/afm157

[22] Braun V, Clarke V. Using thematic analysis in psychology. Qual Res Psychol 2006; 3: 77–101.

[23] Braun V, Clarke V. Reflecting on reflexive thematic analysis. Qual Res Sport Exerc Heal 2019; 11: 589–97.

[24] Delbaere K, Mikolaizak AS, Close JCT, Brodaty H, Lord SR, Sachdev PS. The Falls Efficacy Scale International (FES-I). A comprehensive longitudinal validation study. Age Ageing 2010; 39: 210–6. 20061508 10.1093/ageing/afp225

[25] Dollard J, Barton C, Newbury J, Turnbull D. Falls in old age: a threat to identity. J Clin Nurs 2012; 21: 2617–25. 22393883 10.1111/j.1365-2702.2011.03990.x

[26] Pohl P, Sandlund M, Ahlgren C, Bergvall-Kåreborn B, Lundin-Olsson L, Wikman AM. Fall risk awareness and safety precautions taken by older community-dwelling women and men—a qualitative study using focus group discussions. PLoS One 2015; 10: e0119630. 25781181 10.1371/journal.pone.0119630 PMC4363490

[27] Ellmers TJ, Cocks AJ, Young WR. Evidence of a link between fall-related anxiety and high-risk patterns of visual search in older adults during adaptive locomotion. J Gerontol A 2020; 75: 961–7. 10.1093/gerona/glz176 PMC7164535 31362302

[28] Wong W-L, Masters RSW, Maxwell JP, Abernethy B. The role of reinvestment in walking and falling in community-dwelling older adult. J Am Geriatr Soc 2009; 57: 920–2. 19470013 10.1111/j.1532-5415.2009.02228.x

[29] American Psychological Association . Panic. APA Dictionary of Psychology. https://dictionary.apa.org/panic (10 August 2021, date last accessed).

[30] Delbaere K, Sturnieks DL, Crombez G, Lord SR. Concern about falls elicits changes in gait parameters in conditions of postural threat in older people. J Gerontol A Biol Sci Med Sci 2009; 64A: 237–42. 10.1093/gerona/gln014 PMC2655012 19196645

[31] Wells A . A cognitive model of generalized anxiety disorder. Behav Modif 1999; 23: 526–55. 10533439 10.1177/0145445599234002

[32] Weiner B . An attributional theory of achievement motivation and emotion. Psychol Rev 1985; 92: 548–73. 3903815

[33] Ruthig JC, Chipperfield JG, Perry RP, Newall NE, Swift A. Comparative risk and perceived control: implications for psychological and physical well-being among older adults. J Soc Psychol 2010; 147: 345–69. 10.3200/SOCP.147.4.345-369 17955748

[34] Young WR, Ellmers TJ, Kinrade NP, Cossar J, Cocks AJ. Re-evaluating the measurement and influence of conscious movement processing on gait performance in older adults: development of the gait-specific attentional profile. Gait Posture 2020; 81: 73–7. 32683216 10.1016/j.gaitpost.2020.07.008

