# Supplementary material for: Protective or harmful? A qualitative exploration of older people’s perceptions of worries about falling
Source: Age Ageing. 2022 Apr 1;51(4):afac067. doi: 10.1093/ageing/afac067 (PMC8972997; doi:10.1093/ageing/afac067)
Supplement: aa-21-1751-File003_afac067 [file aa-21-1751-file003_afac067.docx]

**Protective or harmful? A qualitative exploration of older people’s perceptions of worries about falling**

**Appendix 1- Interview guide.**

Thank you for your time and agreeing to talk to me about your experiences and for me to record this interview. I’m part of a research team investigating worries about falling that you may have. We would like to learn how these worries develop, if and how they affect you, and what other consequences you think that they have. All information you provide me will remain anonymous. If you would like to pause the interview at any stage, let me know and if you would like to stop the interview at any stage, that is fine.

**1. Experiences with worrying**

- To start with, I’d like to know more about the worries you have about falling. Can you tell me a little bit more about the worries that you have?
- Are there any specific tasks or situations that you are particularly worried about, or find yourself worrying in? Why? (*Anticipation or during a task/scenario?*)
- If yes, imagine yourself performing this task/ in this situation: What specific worries would be running through your head? How would these be making you feel? Would these be affecting your behaviour?
- What exactly is it that you are worried about? (*How likely are these to happen?*)
- When did these worries first begin?
- What led you to start worrying?
- What do you feel makes your worries worse?

**2. Consequences of worries**

- Thinking about the worries you have identified, can you describe how they have impacted your life?
- What consequences do you think these worries have had for you?
- How do these worries make you feel?
- Do these worries influence the activities you choose to do?
- If they talk about avoiding: What activities? How does avoiding these activities make you feel? What do you think would happen if you were to engage in these?
- *Do they focus on activities that they can – or can’t do?*
- Do you think that these worries affect your balance/walking?

**3. Compensatory strategies**

- Is there anything that you do to try and overcome or reduce these worries?
- Can you think of any times where you had been worrying about falling, but then you did something that helped reduce the worries? If so, what was it that helped?
- Can you tell me about anything else that has been successful in reducing your worries?
- What would managing these worries look like for you?
- If you could get rid of these worries, would you?
- Have you ever discussed these worries with anyone informally (friend or family) or formally (eg GP)?
- If so, what were the outcomes?
- If not, can you talk to me about the reasons why you haven’t?

**4. Free comments**

- Do you have anything else that you would like to say about your experiences?

**Appendix 2- Coding list and development of themes/subthemes.**

**Codes**

- Awareness of balance limitations
- Awareness of growing older
- Previous experience of falling
- Previous experience of losing balance (but without falling)
- Conscious strategies to maintain balance
- Enhanced focus on the task
- Assessing the risk of falling
- Worries about an injury
- Worries about a changing life
- Worries about being unable to stand back up (after a fall)
- Worries as a disruptor
- Activity avoidance
- Losing control
- Feelings of panic
- Loss of confidence
- Self-presentational concerns
- Resilience as a coping mechanism for worry
- Peer-discussions as a coping mechanism for worry
- External device for support

**Preliminary Themes/Subthemes**

**Theme 1:** *“The age of falling”: Awareness of the ageing body*

- Recognition of susceptibility for falling
- Recognition of susceptibility for harm

**Theme 2:** *“A controlled worry”: Worries as a protector*

- Weighing up the risks
- “It’s like an army campaign”: Pre-emptive risk reduction
- Conscious control of balance and walking

**Theme 3:** *Uncertain and out of control: Worries as a disruptor*

- Disruptions to balance
- Disruptions to mental wellbeing
- Disruptions to life

**Theme 4:** *“A prisoner in the house”: Strategies to overcome worries*

- A desire to maximise living
- Generational resilience
- Discussions with peers

**Intermediate Themes/Subthemes**

**Theme 1:** *“The age of falling”: Awareness of the ageing body*

- Recognising susceptibility for falling
- Recognising susceptibility for harm

**Theme 2:** *“A controlled worry”: Worries as a protector*

- Weighing up the risks
- Pre-emptive reduction of risk
- Conscious control of balance and walking

**Theme 3:** *Uncertain and out of control: Worries as a source of panic*

- Disruptions to balance
- Disruptions to mental wellbeing
- Disruptions to life

**Theme 4:** *“A prisoner in the house”: Overcoming worries through resilience*

**Final Themes/Subthemes:**

**Theme 1:** “The age of falling”: Recognition of the ageing body

**Theme 2:** In control of being careful: Worries as a protector

- Identifying the risks and planning for safety
- Consciously engaging movement strategies

**Theme 3:** Uncertain and out of control: Worries as a source of panic

**Theme 4:** “A prisoner in the house”: Activity curtailment and an altered sense of self
